# ChatGPT-4 Performance on German Continuing Medical Education—Friend or Foe (Trick or Treat)? Protocol for a Randomized Controlled Trial

**DOI:** 10.2196/63887

**Published:** 2025-02-06

**Authors:** Christian Burisch, Abhav Bellary, Frank Breuckmann, Jan Ehlers, Serge C Thal, Timur Sellmann, Daniel Gödde

**Affiliations:** 1 State of North Rhine-Westphalia Regional Government Düsseldorf Leibniz-Gymnasium Essen Germany; 2 Department of Didactics and Education Research in the Health Sector Faculty of Health Witten/Herdecke University Witten Germany; 3 Faculty of Health Witten/Herdecke University Witten Germany; 4 Department of Cardiology, Pneumology, Neurology and Intensive Care Medicine Klinik Kitzinger Land Kitzingen Germany; 5 Department of Cardiology and Vascular Medicine West German Heart and Vascular Center Essen University Duisburg-Essen Essen Germany; 6 Department of Anesthesiology HELIOS University Hospital Wuppertal Germany; 7 Department of Anaesthesiology I Witten-Herdecke University Witten Germany; 8 Department of Anesthesiology and Intensive Care Medicine Evangelisches Krankenhaus Hospital, BETHESDA zu Duisburg Duisburg Germany; 9 Department of Pathology and Molecular Pathology HELIOS University Hospital Wuppertal, University Witten/Herdecke Witten Germany

**Keywords:** ChatGPT, artificial intelligence, large language model, postgraduate education, continuing medical education, self-assessment program

## Abstract

**Background:**

The increasing development and spread of artificial and assistive intelligence is opening up new areas of application not only in applied medicine but also in related fields such as continuing medical education (CME), which is part of the mandatory training program for medical doctors in Germany. This study aimed to determine whether medical laypersons can successfully conduct training courses specifically for physicians with the help of a large language model (LLM) such as ChatGPT-4. This study aims to qualitatively and quantitatively investigate the impact of using artificial intelligence (AI; specifically ChatGPT) on the acquisition of credit points in German postgraduate medical education.

**Objective:**

Using this approach, we wanted to test further possible applications of AI in the postgraduate medical education setting and obtain results for practical use. Depending on the results, the potential influence of LLMs such as ChatGPT-4 on CME will be discussed, for example, as part of a SWOT (strengths, weaknesses, opportunities, threats) analysis.

**Methods:**

We designed a randomized controlled trial, in which adult high school students attempt to solve CME tests across six medical specialties in three study arms in total with 18 CME training courses per study arm under different interventional conditions with varying amounts of permitted use of ChatGPT-4. Sample size calculation was performed including guess probability (20% correct answers, SD=40%; confidence level of 1–α=.95/α=.05; test power of 1–β=.95; *P*<.05). The study was registered at open scientific framework.

**Results:**

As of October 2024, the acquisition of data and students to participate in the trial is ongoing. Upon analysis of our acquired data, we predict our findings to be ready for publication as soon as early 2025.

**Conclusions:**

We aim to prove that the advances in AI, especially LLMs such as ChatGPT-4 have considerable effects on medical laypersons’ ability to successfully pass CME tests. The implications that this holds on how the concept of continuous medical education requires reevaluation are yet to be contemplated.

**Trial Registration:**

OSF Registries 10.17605/OSF.IO/MZNUF; https://osf.io/mznuf

**International Registered Report Identifier (IRRID):**

PRR1-10.2196/63887

## Introduction

ChatGPT-4 is the latest development in the large language model (LLM) family from ChatGPT. It is said to be trained on more than one trillion parameters, making it one of the most advanced LLMs currently available for generating conversation-style responses to user input. The parameters are the numerical values that determine how a neural network processes input data and produces output data. They are learned from data during the training process, encoding the model’s knowledge and skills [1].

Since its launch by OpenAI, the ChatGPT family has stimulated widespread conversation and momentum across different specialties in medicine, as demonstrated by more than 3300 publications related to ChatGPT (or Chat-GPT) indexed in PubMed as of mid-May 2024. Generally, LLMs enable humans to interact and discuss a broad range of topics with artificial intelligence (AI) chatbots. New features of ChatGPT-4 include the acceptance of images as input and the generation of captions, classifications, and analyses, which were not available in earlier versions. Compared to its predecessors, ChatGPT-4 is 82% less likely to respond to inappropriate content requests and 40% more likely to provide factual answers than GPT-3 in internal evaluations [2].

After its success in passing the United States Medical Licensing Examination, performing at a level comparable to that of a third-year medical student [3], these results have been largely confirmed across various medical specialties. Comparisons with different ChatGPT versions and other LLM providers, such as Google, support these findings [3-13]. There are currently two reviews on this topic [14,15], and recent data specific to Germany have been published [16].

In conclusion, there is now a range of data on the use of LLMs in undergraduate education and teaching but less on postgraduate education, such as continuing medical education (CME) [17] or self-assessment programs [4]. Table 1 shows a selection of studies on this topic.

In Germany, CME, which is mandatory for medical specialists, requires earning 250 training points over five years. These CME points can be acquired through further specialization, attending congresses and conferences, and studying medical literature with consecutive answers to specific questions in the text. This approach essentially “credits” the self-study time spent attentively reading a text and then answering questions.

For this study, we decided to use ChatGPT-4 because of its extensive database and ability to enter texts directly. Additionally, the input and operation of ChatGPT are carried out by adult high school students without any prior medical training. This was to differentiate as clearly as possible whether and to what extent LLMs can offer support today. This study aims to provide further insight into whether AI, in the form of LLMs, can support various levels of medical education by correctly answering CME-relevant questions, allowing participants to generate CME credits independently of existing medical knowledge.

**Table 1:** Data on ChatGPT and postgraduate medical education (continuing medical education or self-assessment programs).

First author	Year	Study	Comparator	Major findings	Conclusions
Sherazi and Canes [4]	2023	Comparative trial	ChatGPT-3.5 versus ChatGPT-4	GPT-4 scored significantly higher than GPT-3.5 on the AUA^a^ SASP^b^ examinations in overall performance, across all test years, and in various urology topic areas.	Results suggest improvement in evolving AI^c^ LLM^d^ in answering clinical urology questions. Certain aspects of medical knowledge and clinical reasoning remain challenging for LLM.
Riedel et al [16]	2023	Comparative trial	Performance of ChatGPT on OB/GYN^e^ course examinations versus questions from the German medical state licensing examinations	ChatGPT demonstrated consistent and comparable performance across both datasets, providing correct responses at a rate comparable with that of medical students.	ChatGPT has promise as a supplementary tool in medical education and clinical practice, providing efficient and personalized learning experiences and assistance for health care providers.
Noda et al [18]	2024	Comparative trial	ChatGPT-3.5 versus ChatGPT-4 versus Bard (Gemini)	GPT-3.5 and Bard performed similarly while being significantly surpassed by GPT-4. GPT-4’s performance was between third- and fourth-year nephrology residents.	GPT-4 outperformed GPT-3.5 and Bard, meeting the Nephrology Board renewal standards in specific years, albeit marginally. The results highlight LLMs potential and limitations.
Ali et al [6]	2023	Comparative trial	ChatGPT-3.5 versus ChatGPT-4 versus user average	GPT-4 significantly outperformed question bank users and GPT-3.5. Increased word count and higher-order problem-solving were associated with lower accuracy for GPT-3.5 not however for GPT-4.	LLMs achieved passing scores on a mock 500-question neurosurgical written board examination, with GPT-4 significantly outperforming ChatGPT.
Watari et al [13]	2023	Comparison study	Chat GPT-4 versus Japanese Residents’ performance on GM-ITE^f^	Of 137 GM-ITE questions in Japanese, GPT-4 scores were significantly higher than the mean scores of residents.	GPT-4 demonstrated a tendency to score higher on difficult questions. However, GPT-4 scored comparatively lower on questions testing attitudes toward patients and professionalism requiring an understanding of context and communication.
Guerra et al [5]	2023	Comparative trial	ChatGPT-4 versus ChatGPT, SANS^g^ users, medical students, and neurosurgery residents	GPT-4 outperformed ChatGPT exceeding the performances of medical students, neurosurgery residents, and the national average of SANS users across all categories.	GPT-4 significantly outperformed medical students, neurosurgery residents, and the national average of SANS users.

^a^AUA: American Urological Association.

^b^SASP: self-assessment study program.

^c^AI: artificial intelligence.

^d^LLM: large language model.

^e^OB/GYN: obstetrics and gynecology.

^f^GM-ITE: General Medicine In-Training Examination.

^g^SANS: self-assessment in neurosurgery examination.

## Methods

### Study Design

This is a randomized controlled trial that will be undertaken following the Declaration of Helsinki principles and after approval by the local Ethics Committee of Witten/Herdecke University (S-108/2024, date of approval May 15, 2024) and after registration in a study register (open scientific framework). To obtain representative, comprehensive, and meaningful data, three large German publishing houses (Deutscher Ärzteverlag GmbH, Cologne; Georg Thieme Verlag KG, Stuttgart; and Springer Medizin Verlag, Heidelberg) that offer journals with CME will be evaluated for one volume each of already expired journals (ie, without the possibility of earning credit points) from the fields of internal medicine, surgery, gynecology, pediatrics, neurology, and anesthesiology. The CME tests provided by the publishing houses needed no further adaptation for utilization in the study.

### Ethical Considerations

The study protocol which was submitted to and accepted by the Ethics Committee of University Witten/Herdecke (S-108/2024, date of approval May 15, 2024) stated decisively that no monetary or other compensation was to occur. The participant’s information was depersonalized by means of not collecting any personal information to begin with, as they were deemed insignificant to the study’s results leading to participants enrolling entirely voluntarily. Furthermore, to participate in the study, the students were required to provide a signed informed consent form which all students were given the opportunity to voice concerns and questions and were informed about their ability to revoke their consent without having to provide a reason, while not having to expect any repercussions.

### Study Participants

To minimize any influence from prior medical knowledge on the results, adult high school students from North Rhine-Westphalia who are willing to participate will be randomized into three study arms. As high school students can be assumed to be void of relevant medical prior education, the choice to include this group as participants is based on its ability to represent the approximate level of medical knowledge of the general population. The inclusion and exclusion criteria are shown in Textbox 1. It should be emphasized that it is not the high school students who are the participants of the study but the CME course tests to which the methods of the three study arms are applied. Careful considerations were put into the selection of the CME tests screening for images of diseases that students may have deemed disturbing. Such CMEs were excluded. The students were encouraged to voice concerns if the contents of the CME tests resulted in uneasiness. Furthermore, ample opportunity was provided for discussing the students’ experiences if deemed necessary by the participants. All these measures aided in minimizing the potential psychological impact of participation in the study. The experience of the participants while attempting to solve the CME tests is not represented in the study’s results as they were deemed to exceed the scope of the study. The student’s proficiency in the German language was required in the hope of avoiding skewed results based on linguistic incomprehension of the provided literature. The students act purely as “tools,” preferably without any medical knowledge of their own.

**Inclusion and exclusion criteria.**

**Inclusion criteria**

- Aged 18 years or older
- Voluntary participation
- No financial compensation
- German as a native language or at a native speaker level
- Ability to operate ChatGPT or other required software

**Exclusion criteria**

- Being underage
- Refusal to participate
- Insufficient German language skills
- Inability to operate ChatGPT or other required software

### Interventions

This trial is designed as a three-armed randomized controlled trial, including one control arm.

#### Intervention Group 1: “All-In”

In this group, CME-subject-specific text is entered into ChatGPT-4 first, and then, the questions are answered by ChatGPT based on that input.

#### Intervention Group 2: “Just Answers”

In this group, questions are answered using only the knowledge available in the ChatGPT-4 database at that time, without entering any subject-specific text first.

#### Control Group 3: “Search and Find”

Participants in this group were asked to answer the questions using only keywords and common sense without any AI support. As the CME were viewed as digital files, the use of “find in text” of the respective document viewing software was permitted.

The approach to having the CME test questions answered in three study arms aims to uncover significant differences in ChatGPT-4’s ability to solve the CME tests, as well as assess the varying time required in the individual study arms.

### Data Analysis

Sample size calculation was performed for the hypothesis that CME test results will improve from guess probability (20% correct answers, SD= 40%) to pass level (70% correct answers) with a confidence level of 1–α=.95, that is, α=.05, and a test power of 1–β=.95. Therefore, for an independent-samples, two-sided study, a sample size of at least 18 CME training courses per study arm is needed. Nonetheless, since the CME courses, as our study subjects, can be processed repeatedly using different methods without altering them or the methods, a paired-sample study is possible and preferable. This allows the same CME courses to be used in the three study arms. Although a sample size of 9 for a single-sided paired-sample study would be sufficient (only an improvement in CME test results is expected and desired by applying the study arm methods), we decided to take a conservative route and will work with 18 CME tests that will be run through all three study arms. The CME tests treated were randomly chosen from a large number of available tests. Care was taken to ensure that every high school student completed the same number of tests in each arm of the study so that confounding variables in the students were evenly distributed across the three groups. In addition, none of them would work on a single CME test twice using different methods to avoid learning effects. Together with the reuse of the CME tests in the three study arms (paired study, see above), these measures prevent any bias arising from the high school students as performing tools or the CME tests themselves as study participants. The AI-supported arms would run through within the shortest possible time and in the correct order so that unwanted training of the AI or the students during the process could be ruled out as much as possible. Statistical data analysis will be performed using the open-source software “R” (R Core Team; 2023). The data is presented as mean ± SD. Since the aim of the study is to identify differences in the percentage test results due to the different approaches in the three study arms, the Student *t* test (1-tailed) or the Mann-Whitney *U* test will be used for the pairwise comparison of means between groups, depending on the normality of the datasets. The Shapiro-Wilk test will be used to check normality. Fisher’s exact test was used to assess the independence of categorical variables. Benjamini-Hochberg adjustments of *P* values will be applied in multiple comparisons. Values of *P*<.05 were considered statistically significant.

## Results

As of October 2024, we have tested five out of the six students we deemed necessary to examine the 18 CME tests across the three study arms. We are set to terminate the data acquisition by November 2024. The ensuing data analysis is predicted to end in December 2024, enabling us to present our results as early as early 2025.

## Discussion

### Principal Findings

To present the influence of AI as objectively as possible, we deliberately refrained from using medically trained test participants. This allowed us to identify the pure influence of AI. Depending on the degree of success, it was necessary to determine to what extent AI can be permissible in CME training courses and what conditions or protective measures were to be imposed.

Since its launch in 2023, not only the development of ChatGPT but also its integration into the medical context increased rapidly, as shown by the ever more extensive database versions and the steadily growing number of medical publications [3-6,19-23]. However, as the capabilities of AI increase, so does the responsibility of actual intelligence to use it in the best possible way for the benefit of all without causing harm (“primum nihil nocere”). Even if the use of AI to solve examinations, whether student or specialist examinations, has already been investigated several times, there is still a knowledge gap in postgraduate teaching and its effects. Ethical, social, and above all, legal aspects, also need to be clarified.

AI has the potential to revolutionize various aspects of our lives. In medicine, its strength lies in its wide range of possible applications. However, the use of AI in education, training, and specialization must be clearly labeled, as it presents not only opportunities but also weaknesses and threats, particularly with the use of LLMs such as ChatGPT and related programs [1]. The correct use of AI in postgraduate medical education, especially LLMs, still needs to be explored and discussed.

This study aims to show that medical training tests can be successfully completed by medical laypersons using AI, which raises questions about the continued usefulness of current training programs, potential regulations to prevent misuse, and opportunities to harness AI capabilities in this context. Currently, self-study to obtain CME credits is an integral part of German postgraduate medical education. This study’s results may have the potential to influence this practice significantly.

The decision to examine ChatGPT’s results in three study arms was based on the hope of being able to compare the results obtained while gaining insight into whether the literature backgrounds of the CME were required to generate significantly superior results.

The rationale behind the choice of medical specialties whose CME tests were analyzed in the study results from ChatGPT-4’s ability to merely process text. All the medical specialties we chose to include do not predominantly diagnose based on visual symptoms. However, a new investigation on newer ChatGPT versions, which possess the ability to obtain information from images is underway.

### Limitations

This study has several limitations. First, only German CMEs are evaluated. Second, only a minority of specialties were chosen. Finally, we completely dispensed CME with image content, as is usual in radiology, dermatology, or pathology, for example, to avoid changing the selectivity.

How potential biases that result from the data that was used in training the AI tools impacted the results of the study is not derivable, as ChatGPT does not possess knowledge in the classical sense. It is rather the case that lexical data is produced based on prior training.

If knowledge acquired through AI-generated literature is retained differently than medically conventionally attained knowledge is yet to be assessed in future research.

### Conclusions

The impact on current on future CME programs should be considered as the certified means of personalizing one’s medical education remains scarce. AI could play a role in tailoring continuous education to personalized needs and, for example, adapting the modules based on prior results to target potential individual shortcomings. The role AI could play in medical education provided by university faculties, as well as personalized learning programs surely merits further investigation [24-26].

## Abbreviations

AI: artificial intelligence
CME: continuing medical education
LLM: large language model

## Data Availability

The datasets generated during or analyzed during this study are not yet publicly available as they are yet to be analyzed but can be made available from the corresponding author upon reasonable request.

## References

[1] Gödde D, Nöhl S, Wolf C, Rupert Y, Rimkus L, Ehlers J, Breuckmann F, Sellmann T (2023). A SWOT (strengths, weaknesses, opportunities, and threats) analysis of ChatGPT in the medical literature: concise review. J Med Internet Res.

[2] ChatGPT-4.

[3] Gilson A, Safranek CW, Huang T, Socrates V, Chi L, Taylor RA, Chartash D (2023). How does ChatGPT perform on the United States Medical Licensing Examination (USMLE)? The implications of large language models for medical education and knowledge assessment. JMIR Med Educ.

[4] Sherazi A, Canes D (2023). Comprehensive analysis of the performance of GPT-3.5 and GPT-4 on the American Urological Association self-assessment study program exams from 2012-2023. Can Urol Assoc J.

[5] Guerra GA, Hofmann H, Sobhani S, Hofmann G, Gomez D, Soroudi D, Hopkins BS, Dallas J, Pangal DJ, Cheok S, Nguyen VN, Mack WJ, Zada G (2023). GPT-4 artificial intelligence model outperforms ChatGPT, medical students, and neurosurgery residents on neurosurgery written board-like questions. World Neurosurg.

[6] Ali R, Tang OY, Connolly ID, Sullivan PLZ, Shin JH, Fridley JS, Asaad WF, Cielo D, Oyelese AA, Doberstein CE, Gokaslan ZL, Telfeian AE (2023). Performance of ChatGPT and GPT-4 on neurosurgery written board examinations. Neurosurgery.

[7] Ali R, Tang OY, Connolly ID, Fridley JS, Shin JH, Sullivan PLZ, Cielo D, Oyelese AA, Doberstein CE, Telfeian AE, Gokaslan ZL, Asaad WF (2023). Performance of ChatGPT, GPT-4, and Google bard on a neurosurgery oral boards preparation question bank. Neurosurgery.

[8] Khan AA, Yunus R, Sohail M, Rehman TA, Saeed S, Bu Y, Jackson CD, Sharkey A, Mahmood F, Matyal R (2024). Artificial intelligence for anesthesiology board-style examination questions: role of large language models. J Cardiothorac Vasc Anesth.

[9] Hofmann HL, Guerra GA, Le JL, Wong AM, Hofmann GH, Mayfield CK, Petrigliano FA, Liu JN (2024). The rapid development of artificial intelligence: GPT-4's performance on orthopedic surgery board questions. Orthopedics.

[10] Lum ZC (2023). Can artificial intelligence pass the American Board of Orthopaedic Surgery Examination? Orthopaedic residents versus ChatGPT. Clin Orthop Relat Res.

[11] Massey PA, Montgomery C, Zhang AS (2023). Comparison of ChatGPT-3.5, ChatGPT-4, and orthopaedic resident performance on orthopaedic assessment examinations. J Am Acad Orthop Surg.

[12] Yu P, Fang C, Liu X, Fu W, Ling J, Yan Z, Jiang Y, Cao Z, Wu M, Chen Z, Zhu W, Zhang Y, Abudukeremu A, Wang Y, Liu X, Wang J (2024). Performance of ChatGPT on the Chinese postgraduate examination for clinical medicine: survey study. JMIR Med Educ.

[13] Watari T, Takagi S, Sakaguchi K, Nishizaki Y, Shimizu T, Yamamoto Y, Tokuda Y (2023). Performance comparison of ChatGPT-4 and Japanese medical residents in the general medicine in-training examination: comparison study. JMIR Med Educ.

[14] Sumbal A, Sumbal R, Amir A (2024). Can ChatGPT-3.5 pass a medical exam? A systematic review of ChatGPT's performance in academic testing. J Med Educ Curric Dev.

[15] Kim TW (2023). Application of artificial intelligence chatbots, including ChatGPT, in education, scholarly work, programming, and content generation and its prospects: a narrative review. J Educ Eval Health Prof.

[16] Riedel M, Kaefinger K, Stuehrenberg A, Ritter V, Amann N, Graf A, Recker F, Klein E, Kiechle M, Riedel F, Meyer B (2023). ChatGPT's performance in German OB/GYN exams—paving the way for AI-enhanced medical education and clinical practice. Front Med (Lausanne).

[17] Seetharaman R (2023). Revolutionizing medical education: can ChatGPT boost subjective learning and expression?. J Med Syst.

[18] Noda R, Izaki Y, Kitano F, Komatsu J, Ichikawa D, Shibagaki Y (2024). Performance of ChatGPT and Bard in self-assessment questions for nephrology board renewal. Clin Exp Nephrol.

[19] Flores-Cohaila JA, García-Vicente A, Vizcarra-Jiménez SF, De la Cruz-Galán JP, Gutiérrez-Arratia JD, Torres BGQ, Taype-Rondan A (2023). Performance of ChatGPT on the peruvian national licensing medical examination: cross-sectional study. JMIR Med Educ.

[20] Meo SA, Alotaibi M, Meo MZS, Meo MOS, Hamid M (2024). Medical knowledge of ChatGPT in public health, infectious diseases, COVID-19 pandemic, and vaccines: multiple choice questions examination based performance. Front Public Health.

[21] Meo SA, Al-Masri AA, Alotaibi M, Meo MZS, Meo MOS (2023). ChatGPT knowledge evaluation in basic and clinical medical sciences: multiple choice question examination-based performance. Healthcare (Basel).

[22] Ghosh A, Jindal NM, Gupta VK, Bansal E, Bajwa NK, Sett A (2023). Is ChatGPT's knowledge and interpretative ability comparable to first professional MBBS (Bachelor of Medicine, Bachelor of Surgery) students of India in taking a medical biochemistry examination?. Cureus.

[23] R: a language and environment for statistical computing. R Foundation for Statistical Computing.

[24] Steffen V, Nils U, Henner G, Kristina H, Stefan D, Torsten E, Luis L, Alexander M, Maximilian R, Caroline R, Manfred S, Mareike S (2023). Unlocking the power of generative AI models and systems such as GPT-4 and ChatGPT for higher education: a guide for students and lecturers. Hohenheim Discussion Papers in Business, Economics and Social Sciences, No. 02-2023, Universität Hohenheim, Fakultät Wirtschafts- und Sozialwissenschaften, Stuttgart.

[25] Hang CN, Wei Tan C, Yu P (2024). MCQGen: a large language model-driven MCQ generator for personalized learning. IEEE Access.

[26] Fuchs K (2023). Exploring the opportunities and challenges of NLP models in higher education: is Chat GPT a blessing or a curse?. Front. Educ.

